# Cigarette Packs With URLs Leading to Tobacco Company Websites: Content Analysis

**DOI:** 10.2196/15160

**Published:** 2020-06-09

**Authors:** Caitlin Victoria Weiger, Katherine Smith, Amy Y Hong, Joanna E Cohen

**Affiliations:** 1 Institute for Global Tobacco Control Department of Health, Behavior and Society Johns Hopkins Bloomberg School of Public Health Baltimore, MD United States; 2 Department of Epidemiology Johns Hopkins Bloomberg School of Public Health Baltimore, MD United States

**Keywords:** advertising, developing countries, product packaging, product labeling, tobacco industry

## Abstract

**Background:**

Tobacco companies include on the packaging of their products URLs directing consumers to websites that contain protobacco messages. Online media tend to be underregulated and provide the industry with an opportunity to present users with protobacco communication.

**Objective:**

The objective of our study was to document the content of websites that were advertised on tobacco packs in 14 low- and middle-income countries.

**Methods:**

We purchased tobacco packs from 14 low- and middle-income countries in 2013 and examined them for the presence of URLs. We visited unique URLs on multiple occasions between October 1, 2016 and August 9, 2017. We developed a coding checklist and used it to conduct a content analysis of active corporate websites to identify types of protobacco communication. The coding checklist included the presence of regulatory controls and warnings, engagement strategies, marketing appeals (eg, description of product popularity, luxury/quality, taste), corporate social responsibility programs, and image management. We coded brand websites separately and also described social media and other website types.

**Results:**

We identified 89 unique URLs, of which 54 were active during the search period. We assessed 26 corporate websites, 21 brand websites, 2 nontobacco websites, and 5 social media pages. We excluded 2 corporate websites and 14 brand websites due to limited accessible content or incomplete content. Corporate social responsibility was discussed on all corporate websites, and marketing appeals were also common. Corporate websites were also more likely to include more nonspecific (12/24, 50%) than specific (7/24, 29%) health warnings. Promotions (6/7, 86%) and sociability appeals (3/7, 43%) were common on brand websites. The small number of social media webpages in our sample used gendered marketing.

**Conclusions:**

URLs appearing on tobacco packs direct consumers to websites where users are exposed to marketing that highlights the “positive” contributions of tobacco companies on corporate websites, and extensive promotions and marketing appeals on brand websites and social media pages. It is essential that marketing regulations become more comprehensive and ban all protobacco communication, a policy that is in line with articles 5.3 and 13 of the World Health Organization Framework Convention on Tobacco Control. For countries that already ban internet tobacco advertising, enforcement efforts should be strengthened. Tobacco companies’ use of URLs on packs may also be compelling for plain packaging advocacy, where all branding is removed from the pack and large graphic health warning labels are the only communication on the tobacco packaging. Future research should consider including tobacco websites in marketing surveillance.

## Introduction

### Background

Tobacco is the most dangerous consumer product in the world, and tobacco product marketing has been recognized as a cause of tobacco use initiation and failed quit attempts for over a decade [1-10]. The internet provides an additional platform for protobacco communication via tobacco industry websites. The importance of these websites is highlighted by the millions of dollars the industry spends on them; for example, US tobacco companies have been increasing spending on internet marketing since the early 2000s, spending US $25.1 million in 2017 on company websites [11,12]. The continually evolving nature of the internet and tobacco industry marketing strategies makes these websites a public health surveillance priority [13].

Tobacco companies use these websites to promote both their product branding and their corporate image. Given the broad public awareness of the harms of smoking and their deceptive tactics, tobacco companies have taken steps to try to counteract negative perceptions by engaging in activities that they describe as serving to build credibility [14]. The industry touts green business practices, publicizes support for what they see as reasonable (one might imagine, restricted) approaches to regulation, acknowledges some harms caused by smoking, and promotes the future of “reduced-harm” products in order to manage their corporate image and normalize tobacco companies’ practices [14-16]. Although normalization of the industry is itself harmful, these activities additionally enhance industry access to policy makers, influence the credibility of industry-proposed policy alternatives, and weaken opposing political constituencies [17,18]. While the industry formerly relied on traditional media and opinion leaders to spread these messages, they now also incorporate online content, such as company websites [16,19-22]. Tobacco companies print URLs to their websites on their product packaging, presumably increasing exposure. URLs on packs link consumers to much more protobacco communication than can otherwise be contained on the pack.

There are several types of tobacco websites, which tend to fall into 3 main categories: corporate [20], brand [23,24], and social media [25]. Corporate websites represent the company as a formal business and present their corporate image to the public [26,27]. Brand websites market specific products through “interactivity, multidirectional communication and meaningful engagement” [24]. Social media pages can be associated with either a company or a brand, or may be the product of consumer-generated content [28]. Tobacco companies can make “pages” or accounts on unaffiliated social networking platforms that people can like or follow. As an example, Dunhill, a brand of British American Tobacco cigarettes, had a page on Facebook in 2009 to promote Dunhill events, including parties at nightclubs, where free Dunhill cigarettes were distributed [29]. Nontobacco websites, websites not visibly associated with a tobacco company, are also sometimes listed on tobacco packs.

Despite its potential to influence both consumer and policy-maker perceptions of the tobacco industry, online tobacco marketing is still relatively new and underregulated [28,30]. Legislation on tobacco marketing rarely mentions internet marketing specifically, leaving the status of what types of marketing are allowed online up to interpretation (Multimedia Appendix 1). It is also unclear whether corporate marketing for specific companies that promotes the company rather than its products is allowed under advertising and marketing bans.

Tobacco control researchers have already started to document activity on tobacco websites. Previous studies on corporate websites have focused on corporate social responsibility (CSR), the provision of limited health information, and image management [20,26,31,32]. Brand websites were found to tout interactive games, contests, event information, and coupons, and even to allow users to select their own music to enjoy while browsing [22,33-36].

The literature on tobacco internet marketing is, however, somewhat limited. Content analyses of corporate websites have focused on samples of US websites or only the websites of the largest multinational companies [20,26,34]. Research has not investigated websites, even for multinational companies, in low- and middle-income countries (LMICs), where 80% of daily smokers live and where tobacco companies are focusing efforts to expand the cigarette market [37,38]. Research on brand websites also tends to be older and limited to a case study approach, and the constantly changing nature of the internet may make some of those findings less relevant today than when they were originally published [22,23,39,40].

### Objective

To help fill these gaps, we conducted a quantitative content analysis of corporate tobacco and brand tobacco websites and described nontobacco websites and social media pages associated with URLs that we found on tobacco packs purchased in 14 LMICs. Our objective was to assess the marketing strategies to which tobacco companies are linking their consumers. This descriptive analysis may inform marketing regulations and further support packaging restrictions and plain packaging initiatives.

## Methods

### Sample

As part of the Tobacco Pack Surveillance System (TPackSS) study [41], 3307 cigarette, kretek (clove cigarettes common in Indonesia) [42], and bidi (hand-rolled cigarettes common in India and Southeast Asia) [43] country-unique packs were systematically purchased from 14 LMICs in 2013 [41]. We recorded each specific URL using pack photos available from TPackSS [44] and in-person examination of the pack when the URL was not legible from photos (n=56). URLs were located on all sides of the pack (front, back, sides, top, and bottom), as well as on the inside of the packs (inside lid and on paper inserts included in the packs). We entered URLs into an internet search to locate the website, and downloaded and saved screenshots of the home page and each main tab of the menu that was one click away from the home page. While this approach did not allow for the assessment of all content on each website, we established a limit, as it was not feasible to follow every link to every page. We visited websites multiple times between October 1, 2016 and August 9, 2017 to maximize the number of active websites in our sample, as it was common for websites to go through cycles of inactivity and activity.

### Procedure

If an English-language version of the website was not provided, we asked a translator to write the English translation of the text on the saved PDFs of website images. Translators were then present (either physically or via Skype; Microsoft Corporation) while coders coded these PDFs in the event that coders had questions about the meaning of the translation. We used codes from a content analysis of Russian e-cigarette websites [45] and common features found on US tobacco websites during field testing to develop a codebook to quantitatively inventory website content. Categories were engagement strategies, marketing appeals, regulatory controls and warnings, and CSR and image management. We considered graphic content only for codes related to flavor and marketing appeals that could be easily and reliably identified (ie, luxury/quality, sociability, masculinity, and femininity appeals). We programmed the coding checklist into Qualtrics software version 05.2017 to 09.2017 (Qualtrics, LLC) to facilitate coding (Multimedia Appendix 2).

Due to high interrater reliability assessed after coding the first 20 websites, not all of the remaining websites were double coded, resulting in 81% (25/31) of quantitatively coded (ie, corporate and brand) websites being double coded. We coded the websites’ home page and each main tab from the website menu for presence versus absence of each feature. We assessed interrater reliability using percentage agreement, as well as a method to account for the low prevalence of some marketing strategies (prevalence-adjusted and bias-adjusted kappa) [46-48]. Overall percentage agreement was .95 and prevalence-adjusted and bias-adjusted kappa was .89. Discrepancies were reviewed and reconciled via in-person discussion between the coders, with clarifications made to the codebook as needed.

### Analysis

Data were exported from Qualtrics into an Excel spreadsheet version 16.6 (Microsoft Corporation) and analyzed using Stata version 15 (StataCorp LP). We calculated the proportion of websites whose content reflected each of the codes.

We assessed brand websites using the same coding checklist used for corporate websites, although we present the results separately due to the limited amount of content visible without registration. We briefly described the social media pages and nontobacco websites, since their characteristics were not adequately captured by the codebook.

## Results

### Website Characteristics

**Figure 1:**
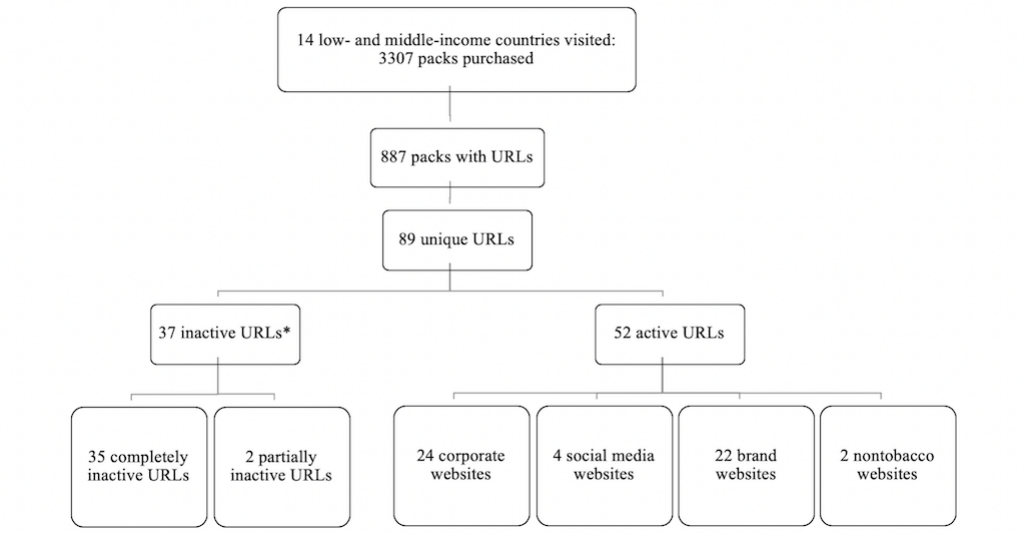
Breakdown of coded websites. *37 websites considered inactive for the following reasons: the page could not be found (n=17), the page took too long to respond (n=7), the site was closed for construction (n=5), the domain name had expired (n=4), some parts of the page were inactive (n=2), the company had been sold (n=1), or access was not allowed (n=1).

We noted the presence and location of an URL on 880 packs (857 cigarette packs and 23 kretek packs). Of these, 7 packs included 2 URLs. There were 54 active and unique websites: 26 were corporate websites, 21 were brand websites, 5 were social media websites, and 2 were nontobacco websites. We excluded 2 corporate websites because not all website pages were active (see Figure 1 for details). All brand websites but 1 required the user to register in order to see complete website content. Registration required local addresses, verification of country citizenship, and verification that you were a tobacco product user. We excluded 14 brand websites with no content visible prior to registration, as we could not breech the terms and conditions of the websites per university requirements.

The 38 websites that were accessible for analysis were from 560 packs, or 63.6% of all 880 purchased packs with URLs. These packs were from 13 countries, and the websites were available in over 20 languages, counting all languages available on each webpage (Multimedia Appendix 3). We found the 24 active corporate websites included in this analysis on 486 packs purchased in 13 countries, or 14.70% of all 3307 purchased packs and 55.2% of all 880 purchased packs with URLs. We categorized 8 websites as multinational and 16 as nonmultinational. In line with work by others, we considered the 4 leading multinational tobacco companies, Philip Morris International, British American Tobacco, Japan Tobacco International, and Imperial Tobacco [49], to be multinational. Other corporate websites were from state-owned and other regional or national companies. The 7 active brand website URLs with at least some content visible were on 52 packs from 6 countries, the 5 social media webpage URLs were on 19 packs from 3 countries, and the 2 nontobacco website URLs were on 3 packs from 2 countries (Multimedia Appendix 3).

### Corporate Websites

#### Regulatory Controls and Warnings

Of the 24 corporate websites, 14 (58%) had statements regarding the legal age for product purchase or website use (Table 1). We found pop-up windows asking whether the user was over the age of 18 years (known as click-through verifications) on 3 (13%) websites, but no website had a specific verification requirement (eg, exact date of birth). Of the 24 websites, 7 (29%) had specific health warnings (eg, from the Turkish Imperial Tobacco website: “Smoking is a cause of serious diseases in smokers, including lung cancer, heart disease and emphysema”), whereas 12 (50%) had only general acknowledgements of the health risks of smoking (eg, from the Tutun company website: “Smoking is negatively impacting your health”).

**Table 1:** Codebook used for corporate (n=24) websites and proportion of corporate websites by the presence of each code.

Domain			Code prevalence on corporate websites, n (%)
**Regulatory controls and warnings**			
	Access restrictions	3 (13)	
	Age-restricted product usage statements	14 (58)	
	General health warning	12 (50)	
	Specific health warning	7 (29)	
**Engagement strategies**			
	Link to additional corporate website (eg, parent website)	7 (29)	
	Link to a brand website	2 (8)	
	Link to a social media page	14 (58)	
	Link to another type of webpage	15 (63)	
	Option to download an app	2 (8)	
	Email alerts	3 (13)	
	Contests	1 (4)	
	Video	9 (38)	
	Scrolling images	15 (63)	
**Marketing appeals**			
	Product popularity	13 (54)	
	Luxury/quality	21 (88)	
	Femininity	1 (4)	
	Masculinity	0 (0)	
	Taste/sensation	10 (42)	
	Flavor other than menthol	6 (25)	
	Menthol flavor	3 (13)	
	International appeal	19 (79)	
	National appeal	19 (79)	
	US appeal	6 (25)	
	Trendsetter	1 (4)	
	Tradition	16 (67)	
	Light	2 (8)	
	Low tar	3 (13)	
	Other harm reduction term	5 (21)	
**CSR^a^ and image management**			
	General mention of CSR	17 (71)	
	Educational support	8 (33)	
	Investment in community	10 (42)	
	Harm reduction technology	5 (21)	
	Provider of jobs	14 (58)	
	Employee care/support	16 (67)	
	Youth smoking prevention program	3 (13)	
	General environmental claim	11 (46)	
	Specific environmental claim	6 (25)	
	Performance or CSR awards	13 (54)	
	Description of consumers as adults	9 (38)	
	Messaging about informed consumers	6 (25)	
	Support for tobacco control legislation	9 (38)	
	Transparency	9 (38)	
	Innovative	19 (79)	

^a^CSR: corporate social responsibility

#### Engagement Strategies

A few corporate websites offered opportunities for further engagement with their company by offering mobile apps (n=2, 8%) and prompting users to sign up for email alerts (n=3, 13%) (Table 1). British American Tobacco’s website had an app for investors to track company performance. British American Tobacco and Imperial Tobacco both offered email alerts for company media coverage. Some websites also linked to other corporate websites, including the parent company (n=7, 29%), brand (n=2, 8%), and social media websites and webpages (n=14, 58%).

#### Marketing Appeals

Every corporate website contained at least one marketing appeal for their products or company (Table 1). Luxury and quality appeals were the most common (n=21, 88%), such as British American Tobacco’s description of its “superior quality next generation products.” National appeals (n=19, 79%) and appeals to company tradition or history (n=16, 67%) were common. Hongta Tobacco Group Co., a Chinese tobacco company, appealed to company tradition by dedicating a page of its website to the history of the company from its founding in 1956 to its “courageous” introduction of advanced foreign technology. We found popularity appeals, such as Altria Group’s boasting of their ownership of Philip Morris USA (“Leading cigarette manufacturing in the U.S. and has been for more than 30 years”) and that of U.S. Smokeless Tobacco Co. (the “World’s leading producer of moist smokeless tobacco”), on 13 (54%) websites. Appeals to the taste or sensation of tobacco were present on 10 (42%) corporate websites. For instance, the Djarum website described how the “exotic flavors of Djarum kretek has captured the imagination of a growing international audience” [50], using both taste or sensation and international appeals. We also observed international appeals (n=19, 79%), including international appeals mentioning American culture or values (n=6, 25%).

#### Corporate Social Responsibility and Image Management

All 24 corporate websites mentioned CSR or a specific CSR program at least once. Examples of CSR were providing scholarships or other academic support (n=8, 33%), caring for employees and providing support (n=16, 67%), being a provider of jobs (n=14, 58%), and using or investing in harm reduction technology (n=5, 21%). The website for British American Tobacco Russia stated that they were one of the best employers in Russia and claimed to offer employees “all possibilities for professional and career growth.” Hongta publicized several educational initiatives on their websites, including a ¥4 million (about US $565,000) donation to a middle school and the completion of a dormitory building project for another school. Youth smoking prevention programs were referenced on 3 (13%) websites: 2 British American Tobacco websites and Hongta’s websites. A total of 10 (42%) websites discussed support for communities, including Japan Tobacco International via their ARISE program that is “bringing an end to child labor by working with tobacco farming communities to create new traditions.” A total of 11 (46%) websites contained general references to environmental sustainability, such as British American Tobacco’s “comprehensive environmental management program” [51]. A further 6 (25%) websites provided specific examples of sustainable or environmentally friendly practices, including Shaanxi’s “energy-saving emission reduction program.” Company corporate image was portrayed via mention of company transparency (n=9, 38%), descriptions of the company as innovative (n=19, 79%), discussion of the importance of consumer education (n=6, 25%), and mention of awards won for performance or CSR activities (n=13, 54%). The Russian company Donskoy Tabak endorsed their “principles of openness and transparency” and showcased their awards for “Quality and Technological Effectiveness.” In addition, 9 (38%) websites expressed support for tobacco control legislation or emphasized compliance with such legislation. For example, Imperial Tobacco stated that “We recognize the controversial nature of our product and believe it is important to have it produced and handled by a responsible business. We both comply with and exceed current regulatory standards as we apply our own corporate standards.”

### Brand Websites

Although we coded only 7 brand websites because the other 14 brand websites in the sample set conditions for access incompatible with our university research standards, there are a few observations of note. Of these 7 websites, 6 limited the content visible without registration, while 1 was totally accessible without registration. Despite the limited content visible without registration, we observed promotions such as games, contests, and sponsored events (n=6, 86%), and youth appeals (n=2, 29%). A total of 3 (43%) brands websites included sociability appeals, which were illustrated on Bond Street’s website through images of a small group of attractive young adults playing a guitar by the water. We noted national appeals, including a Marlboro-sponsored event called M-City, which attempted to express the culture of Kiev through art by inviting artists and musicians to the city, on 5 (71%) of the websites. Images of cities at night were common, and some websites included music. Most websites (n=6, 86%) used video or scrolling images, such as scenes of dance parties and cities at night on the Clas Mild websites and videos of “life hacks” on the L&M website.

### Social Media

Of the 5 social media pages in this sample, 4 required that users request to join the group or register to see all content. Our requests to join were not accepted, so only a small amount of content was visible. The 2 social media pages for Kiss cigarettes were both characterized by femininity appeals, with flowers and text describing the page as a group for “modern girls.” The Style Super Slim Tumblr-style social media page included posts on makeup, nail art, fashion, decorating, and baking. Another social media page for 21 Bek, however, used explicit masculine appeals, calling the page a group for “real men.” Black Xperience also used masculine messaging and was a Tumblr-style website with images of cars, motorcycles, attractive women, and something called the “Black Innovation Challenge.”

### Websites Not Related to Tobacco

Two URLs found on packs led to websites that were not for tobacco companies or for tobacco products. One website was for the Programme for the Endorsement of Forest Certification, which described an agenda of caring for the world’s forests and promoting sustainable forest management. Information on sustainability was available, and there was an option to sign up for newsletters from the organization. The other website was for the Japanese Ministry of Health, and the URL is part of the pack warning label required in Japan, although the 2 packs with this URL in the warning were purchased in Turkey. The Japanese Ministry of Health website contained information about the health hazards posed by tobacco, tobacco cessation, a passive smoke-free society movement, and a discussion of the Japanese Ministry’s involvement with the World Health Organization (WHO) Framework Convention on Tobacco Control (FCTC).

## Discussion

### Principal Findings

URLs on tobacco packs purchased in LMICs led to corporate, brand, social media, and other websites that linked consumers to protobacco communication, including marketing for tobacco products and the companies that produce them. Several findings regarding the low prevalence of specific health warnings, use of CSR and other image management techniques, and use of marketing and engagement strategies highlight the potential of tobacco websites to communicate positive messages about the tobacco industry and tobacco products to users. These findings highlight the importance of tobacco websites in the tobacco marketing landscape in LMICs. There is a need for focused research on tobacco marketing in LMICs, as this is where the majority of tobacco users reside and where the industry has increasingly marketed its deadly products [37,38]. Tobacco websites should be explicitly included in marketing restrictions and regulations to protect the public from protobacco communication.

Corporate websites largely failed to communicate many of the harmful effects of smoking to website users. While half of the websites in this sample had a health warning, most of these warnings described vague risks associated with using tobacco, and risk of death and specific diseases were uncommon. However, having a health warning, even a nonspecific one, could be a way for a company to mitigate its liability [52] while presenting a more positive and responsible face to the public [32].

The emphasis of CSR programs on corporate websites was also of particular concern, as these programs can increase perceptions that the company is socially responsible, liking for the company, and positive associations with the company’s products [53]. CSR has been used as a reputation and impression management tool to gain access to decision makers and increase tobacco company influence [17], and has been found to directly interfere with attempts to denormalize the tobacco industry and its products [54]. The very act of selling cigarettes, an addictive product that kills half its long-term users, is antithetical to CSR [55]. Additionally, corporate websites describe themselves as transparent, responsible, innovative companies that acknowledge the importance of consumer education, support tobacco control legislation, and even receive awards for their performance and CSR efforts. These strategies are used to promote an image of tobacco companies as socially responsible. This image serves their own monetary gain rather than the well-being of current and future tobacco consumers, who would be better served by the tobacco industry abandoning advertising and promotion of their products, and not opposing government regulation that aims to reduce tobacco-caused death and disease [56]. Articles 5.3 and 13 of the FCTC further recommend that parties mitigate the effect of “commercial and other vested interests of the tobacco industry” by denormalizing or regulating tobacco industry CSR or by banning CSR entirely by defining it as a form of sponsorship [57,58].

Corporate websites also promoted youth smoking prevention programs. Industry-funded youth smoking prevention advertising has been associated with increased intentions to smoke [59]. The WHO calls on FCTC parties to decline any industry involvement in tobacco use prevention [60], but our research demonstrated that the industry is still involved in tobacco use prevention online. Efforts should be made to keep the tobacco industry out of all tobacco control initiatives, across all media and both online and offline.

While corporate websites did not have contests, coupons, and games that have been documented on brand websites [22,33,34], corporate websites used other types of engagement strategies such as apps and email alerts. Corporate websites also included additional links to other corporate tobacco websites, brand websites, and social media pages, connecting users with even more protobacco content. This is in line with other recent findings on brand websites, which similarly found that all assessed brand websites contained at least one link to a social media page [34,61]. Tobacco companies used each communication platform left open to them to link to other platforms to further spread their messages.

Although our assessment of brand websites was limited by registration requirements, the high prevalence of promotional material visible even from the registration page is cause for concern. Exposure to promotions is associated with youth and young adult initiation, as well as lower likelihood of smoking cessation [1,62-65]. The use of music, both playing in the background and as part of some promotions, is also concerning given that the industry has previously employed music as a way to target youth and racial and ethnic minorities [66]. Brand websites frequently used sociability appeals and were often portrayed through images of attractive young people gathering with friends. Such marketing may be more likely to reach young people online, where they are increasingly spending more of their time. The limited amount of information we were able to observe on social media webpages indicated that this type of website may be more focused on marketing to specific subgroups, as evidenced by the explicitly gendered appeals found on parts of the websites that we were able to access.

URLs on cigarette packs can link consumers with corporate and brand marketing that in turn may undermine restrictions imposed on the physical marketing environment. Monitoring how the tobacco industry adapts to an increasingly restrictive physical marketing environment through the use of URLs on packs and other innovations could be used to inform increased packaging restrictions and plain packaging to reduce the industry’s ability to link tobacco users with additional protobacco messaging. Future marketing restrictions should explicitly include the online environment, and surveillance efforts should monitor industry websites to ensure proper enforcement of existing marketing restrictions.

### Limitations and Strengths

The codebook for this project assessed a finite number of topics. Additionally, we did not code every webpage, leaving some content undocumented. Given the massive amount of content on websites we would have obtained if we had assessed every possible web component (including both internal and external links), we had to establish a limit on what was possible to code.

A total of 15 websites required translation; while translators were fluent in the translated language and in English, it is possible that there were some inaccuracies. To try to account for any confusion about translation, translators were present either in person or via Skype for clarification. Tobacco packs where the websites were found were purchased in 2013, and at the time of analysis (2016-2017) some websites were inactive, reducing the number of websites in our sample.

We were able to include coding only of corporate websites, nontobacco websites, and a limited number of brand and social media websites due to the terms and conditions regarding who was allowed to register and access website content. This limitation prevented us from deeply exploring brand and social media websites and allowed only a cursory glimpse of the content on these types of websites. There is a need for the research community to recognize and address such restrictions and establish norms for conducting critical public health surveillance research in online spaces.

To our knowledge, this is the most extensive analysis of an international sample of corporate tobacco websites. The sampling frame is unique in that URLs were from cigarette packs purchased in 14 LMICs, therefore covering a broader range of available websites, including state-owned and regional and national company websites in addition to country-specific multinational websites. Research on marketing, specifically internet marketing, in LMICs is rare.

### Conclusions

The very existence of tobacco websites, whether corporate, brand, or social media, challenges existing laws concerning tobacco marketing in the countries where packs with URLs were purchased. According to legal analysis from the Tobacco Control Laws website [67] (see Multimedia Appendix 1), at least 7 of the 13 countries where packs were purchased currently ban online tobacco advertising. Bans in China and Thailand were passed after packs were purchased in 2013, but even these 2 later bans were in place before we conducted our content analysis and should have resulted in these websites being removed. An additional 3 countries have legislation that could be interpreted as banning online advertising, and 2 have some restrictions on online advertising. Brand and social media tobacco website URLs appeared on packs purchased in Russia and Vietnam, where online marketing is banned. It’s unclear whether corporate websites, which clearly include marketing appeals and engagement strategies, as well as the promotion of CSR programs, are included in these bans. What is clear is that tobacco companies are taking advantage of this lack of clarity in legislation and lack of enforcement to use the internet to further disseminate both corporate and brand marketing that serves to improve their corporate image and keep people using tobacco products. It is essential that marketing regulations become more comprehensive and ban all protobacco communication, a policy that is in line with articles 5.3 and 13 of the FCTC [57,58]. Plain packaging and other packaging restrictions would also limit the industry’s ability for mass dissemination of their websites and should also be considered. These policy recommendations are especially important for LMICs, where the tobacco industry has concentrated their marketing efforts to take advantage of the looser regulatory environment [37,68,69]. Efforts to monitor tobacco company marketing should consider including online marketing, as well as social media activities, in light of their growing ubiquity.

## Appendix

Multimedia Appendix 1 Online marketing regulations in 13 low- and middle-income countries where URLs on packs were found.

Multimedia Appendix 2 Codebook for quantitative coding of corporate and brand websites.

Multimedia Appendix 3 List of active URLs and associated pack information.

## Abbreviations

CSR: corporate social responsibility
FCTC: Framework Convention on Tobacco Control
LMICs: low- and middle-income countries
TPackSS: Tobacco Pack Surveillance System
WHO: World Health Organization
