# Supplementary material for: Cigarette Packs With URLs Leading to Tobacco Company Websites: Content Analysis
Source: J Med Internet Res. 2020 Jun 9;22(6):e15160. doi: 10.2196/15160 (PMC7312247; doi:10.2196/15160)
Supplement: Multimedia Appendix 1 [file jmir_v22i6e15160_app1.pdf]

Online marketing regulations in 13 LMICs where URLs on packs were found from TobaccoControlLaws.org[67]

Country	TobaccoControlLaws.org interpretation of presence of internet advertising ban	Relevant Legislation
Brazil	Banned: The law specifically bans "commercial advertising" of tobacco products "by electronic media, including the internet."	Law No. 9.294 (as amended by Law No. 10.167/00, Provisional Measure No. 2.190-34/01, and Law No. 10.702/03)<br>*Arts. 3, 3-A(III)*<br>Law No. 12.546 of December 14, 2011<br>*Art. 49*
Russian Federation	Banned: The law prohibits all forms of tobacco advertising, promotion and sponsorship. The law incorporates the FCTC definition of "tobacco advertising and promotion." Therefore, tobacco advertising and promotion via internet is prohibited.	Federal Law No. 15-FZ of February 23, 2013<br>*Art. 16*
Turkey	Possibly Banned: Article 3(1) of the law prohibits all forms of advertising or promotion of tobacco products by using the name, logo, or trademark of the product or producer. Additionally, Art. 3(5) of the law prohibits all forms of announcements or advertisements of tobacco products in the media using the product name, logo, or trademark, no matter what the purpose may be. These provisions could be interpreted as prohibiting tobacco advertising via internet communications.	The Law on Prevention and Control of Hazards of Tobacco Products (Law No. 4207, title as amended)<br>*Arts. 3(1), (5), (11)*
Pakistan	Allowed: Neither the 2002 Ordinance nor Notification F.13-5/2003 restricts internet communications. The law is, therefore, interpreted as allowing tobacco advertising and promotion via internet communications.	Prohibition of Smoking in Enclosed Places and Protection of Non-smokers Health Ordinance, 2002<br>*Section 7*
Philippines	Banned: The law specifically bans all forms of tobacco advertising in mass media, except at the point of sale. By definition, the ban applies to all internet tobacco advertising.	Tobacco Regulation Act of 2003 - An Act Regulating the Packaging, Use, Sale, Distribution, and Advertisements of Tobacco Products and for Other Purposes, Republic Act No. 9211<br>*Section 22*
China	Banned: The Advertising Law prohibits the publication of tobacco advertisements in mass media, and states that the law	Advertising Law of the People's Republic of China (as amended on April 24, 2015)

	specifically applies to advertising activity using the internet. Therefore, tobacco advertising via internet communications is prohibited.	*Arts. 22, 44*
Bangladesh	Banned: The law prohibits producing or disseminating an advertisement of tobacco products through any means including through electronic media and websites. The definition includes any kind of commercial action with the aim of promoting a tobacco product or tobacco use either directly or indirectly. Therefore, tobacco advertising and promotion is prohibited in internet communications.	Smoking and Using of Tobacco Products (Control) Act, 2005<br>*Art. 5*<br>Smoking and Tobacco Products Usage (Control) (Amendment) Act, 2013 (Act No. 16 of 2013)<br>*Art. 5*
Mexico	Possibly Banned: The law and regulations do not address internet communications specifically, but Art. 23 of GLTC provides that publicity and promotion may only be aimed at adults through adult magazines, personal communication by mail, or within establishments exclusively for adult access. This is interpreted as banning advertising and promotion via internet communications.	General Law on Tobacco Control (Ley General para el Control del Tabaco)<br>*Art. 23*<br>Regulation of the General Law on Tobacco Control (as amended) (Reglamento de la Ley General para el Control del Tabaco)<br>*Art. 40*
Vietnam	Banned: The law strictly prohibits advertising and promotion of tobacco products, direct marketing to the users of tobacco in any form, and encouraging, persuading, and coercing other people to use tobacco. In addition, Decree No. 119 specifically prohibits tobacco advertising and promotion in any form.	Decree No. 119/2007/NĐ-CP on Tobacco Manufacturing and Trading<br>*Art. 39*<br>Law No. 09/2012/QH13, Law on Prevention and Control of Tobacco Harms<br>*Arts. 9.2, 9.9*
Thailand	Banned: The law contains a general ban on tobacco advertising and promotion (Art. 30) and explicitly prohibits tobacco advertising "upon or within printed matter, tapes or other video media, motion pictures, radio broadcasts, television broadcasts, electronic media, computer networks, or advertising signs" (Art. 31). "Internet communications" take place through "electronic media" and "computer networks." Therefore, tobacco advertising is prohibited through internet communications.	Tobacco Products Control Act, 2017<br>*Arts. 30, 31*

India	Possibly Banned: It is unclear whether the definition of "advertisement" encompasses all forms of tobacco advertising and promotion. The law does provide a blanket prohibition on the display of any advertisement of cigarettes or other tobacco products. Further, the law prohibits persons "having control over a medium" to advertise tobacco through such a medium. These provisions appear to ban advertising of tobacco products in internet communications.	Cigarettes and Other Tobacco Products (Prohibition of Advertisement and Regulation of Trade and Commerce, Production, Supply and Distribution) Act, 2003<br>*Secs. 5(1) and (2)*
Ukraine	Some Restrictions: The Law on Advertising prohibits tobacco advertising and promotion on websites, "except for websites intended for adults, where preliminary identification of the age of users is a mandatory condition for access." The law also prohibits tobacco advertising and promotion by email. Because the law permits some tobacco advertising and promotion via internet, the regulatory status code "Some Restrictions" has been assigned.	Law of Ukraine No. 270/96-BP on Advertising, July 03, 1996 (as amended through March 18, 2008)<br>*Arts. 22.1, 22.6*<br>Law No. 3778, On the Introduction of Changes to Some Legislative Acts of Ukraine on the Prohibition of the Advertising, Sponsorship and Promotion of the Sale of Tobacco Products<br>*Sections I.2, I.3*
Indonesia	Some Restrictions: Tobacco advertising via "information technology media" specifically is addressed in the law. Advertisements may not show, among other things, cigarettes, cigarette and other tobacco product packs, the shape of cigarettes, tobacco product branding, or smoking. Age verification programs further must be used to restrict access to individuals over 18.	Government Regulation of the Republic of Indonesia No. 109 of 2012 Concerning Materials that Contain Addictive Substances in Tobacco Products in the Interests of Health<br>*Arts. 27, 30, 39*
