# Supplementary material for: Cigarette Packs With URLs Leading to Tobacco Company Websites: Content Analysis
Source: J Med Internet Res. 2020 Jun 9;22(6):e15160. doi: 10.2196/15160 (PMC7312247; doi:10.2196/15160)
Supplement: Multimedia Appendix 2 [file jmir_v22i6e15160_app2.docx]

Codebook for quantitative coding of corporate and brand websites

| **Domain** | **Definition** |
| --- | --- |
| **Engagement strategies** |  |
| Link to additional corporate website | Includes a link to an additional corporate website (e.g. parent company website) |
| Link to a brand specific websites | Includes a link to a brand-specific website |
| Link to a social media page | Includes a link to an affiliated social media page |
| Link to another type of webpage | Includes a link to another type of website |
| Option to download an app | Text to download an app related to the website content (stock monitoring, employment opportunities, games, etc.) |
| Email alerts | Text to sign up for email alerts for more information for the company |
| Contests | Textual reference to or links to brand- or product-related contests |
| Video | Use of video |
| Scrolling images | Use of multiple still images that scroll or play |
| **Marketing Appeals** |  |
| Product popularity | Textual reference indicates/implies that many people use the product, it is very popular or a market leader |
| Luxury/Quality | Textual or graphic reference to the product as a high-status, high quality, luxury product, associates products with high class, glamour |
| Femininity | Textual or graphic reference to product in association with feminine ideals |
| Masculinity | Textual or graphic reference to product in association with masculine ideals |
| Taste/Sensation | Textual reference to to taste (e.g., rich, full), aroma, or sensation (e.g., cool, fresh, refreshing, smooth) including descriptors on packaging |
| Flavor | Textual or graphic reference to flavor descriptors, includes favor capsules but excluding menthol |
| Menthol flavor | Textual or graphic reference to menthol or mint as a flavor descriptor, includes flavor capsules |
| International appeal | Textual reference to global sales/identity, world leader, etc. |
| National appeal | Textual reference to the company's country/national identity/national focus |
| U.S. appeal | Textual reference to identification of product with American ideals or identity |
| Trendsetter | Textual reference to the product in association with looking “cool,” being a “trendsetter,” not conforming to the status quo, or listening to others |
| Tradition | Textual reference to the company’s long history of business/expertise from experience |
| Light | Use of the specific term "Light" to describe products |
| Low tar | Use of the specific term "Low tar" to describe products |
| Other harm reduction term | Use of other specific terms that connote s hard reduction |
| **Regulatory Controls and Warnings** |  |
| Access restrictions | Mechanism to block website access based on age of user (e.g., pop-up box asking for age verification before permitting entry to site) |
| Age-restricted product usage statements | Textual reference to age-based restrictions in relation to the use of tobacco products or the website (e.g., tobacco can only legally be purchased by those over the age of 18) |
| General health warning | General textual statement that tobacco can be injurious to health but does not mention specific diseases or likelihood of death or disease as a result of using tobacco products |
| Specific health warning | Textual description of specific health-related risks and diseases associated with tobacco product usage |
| **CSR and Image Management** |  |
| General mention of CSR | General textual reference to having a social responsibility team, program, or plan |
| Educational support | Textual reference to funding scholarships or supporting academics |
| Investment in community | Textual reference to investment with or involvement with local community or cultural activities |
| Harm reduction technology | Textual reference to investing in or using harm reduction technology |
| Provider of jobs | Textual reference that the company is a provider of jobs for retailers, farmers, factory workers, etc. |
| Employee care/support | Textual reference that the company cares for the wellbeing of employees, provides advancement opportunities and educational training, etc. |
| Youth smoking prevention program | Links or textual reference to the company having a youth smoking prevention program |
| General environmental claim | General textual descriptor of the company as sustainable, environmentally friendly, or green |
| Specific environmental claim | Textual reference to a specific green business practice is listed (e.g., use of alternative energy, reduced CO2 emissions, green farming practices) |
| Company Awards | Textual reference that the company has received awards for company performance, including CSR activities |
| Description of consumers as adults | Textual description of consumers as adults |
| Messaging about informed consumers | Textual reference to the importance of consumer education or a belief that consumers should be informed of the risk of tobacco product usage |
| Support for tobacco control legislation | Textual reference to support for or compliance with tobacco control legislation (e.g., Country laws) |
| Transparency | Textual reference to transparency, openness or trustworthiness of the company in relation to tobacco products or business practices |
| Innovative | Textual reference to the company as innovative, highly automated, high tech |
