# Supplementary material for: Cigarette Packs With URLs Leading to Tobacco Company Websites: Content Analysis
Source: J Med Internet Res. 2020 Jun 9;22(6):e15160. doi: 10.2196/15160 (PMC7312247; doi:10.2196/15160)
Supplement: Multimedia Appendix 3 [file jmir_v22i6e15160_app3.docx]

List of Active URLs and Associated Pack Information

| **Company/Brand name** | **URL** | **Website description** | **# of packs with URL** | **Country where pack was purchased** | **Language** |
| --- | --- | --- | --- | --- | --- |
| Altria | www.pmusa.com | Corporate: multinational company (pmusa.com goes to Altria website) | 3 | Pakistan, Philippines | English |
| Anhui | http://www.ahycgy.com.cn | Corporate: China National Tobacco Corporation Company | 12 | China | Mandarin |
| British American Tobacco | www.bat.com US & www.tdr.hr | Corporate: multinational company | 27 | Turkey; Pakistan, Bangladesh | English |
| British American Tobacco Turkey | www.bat.com.tr | Corporate: multinational company | 23 | Turkey | Turkish |
| British American Tobacco Mexico | http://www.batmexico.com.mx/ | Corporate: multinational company | 37 | Mexico | Spanish |
| British American Tobacco Russia | www.batrussia.ru | Corporate: multinational company | 71 | Russian Federation | Russian |
| Ciroomex | www.ciroomex.com | Corporate: non-multinational company | 3 | Mexico | Spanish |
| Djarum | www.djarum.com | Corporate: non-multinational company | 6 | Pakistan, Russian Federation | English |
| Dontabak | www.dontabak.ru | Corporate: non-multinational company | 5 | Russian Federation | Russian and English |
| Fujian | www.fjtic.com.cn | Corporate: China National Tobacco Corporation Company | 17 | China | Mandarin |
| Heilongjiang | www.lopato.com.cn | Corporate: China National Tobacco Corporation Company | 2 | China | Mandarin |
| Hongta | www.hongta.com | Corporate: China National Tobacco Corporation Company | 38 | China, Pakistan, Bangladesh | Mandarin, English, Romania, Spanish, Vietnamese, and Lao |
| Hubei | www.hbtobacco.com | Corporate: China National Tobacco Corporation Company | 31 | China | Mandarin |
| Imperial tobacco | www.imperialtobacco.com.tr | Corporate: multinational company | 36 | Turkey | Turkish and English |
| Japanese Tobacco International | www.jti.com | Corporate: multinational company | 83 | Pakistan, Bangladesh, Vietnam, Turkey, Thailand, Russian Federation, India | English |
| Kings royal | www.kingsroyal.net | Corporate: non-multinational company | 2 | India, Bangladesh | English |
| Pogarskaya | www.pccf.ru | Corporate: non-multinational company | 2 | Russian Federation | Russian |
| Shaanxi | www.shaanxizhongyan.com.cn | Corporate: China National Tobacco Corporation Company | 2 | China | Mandarin |
| Souza cruz | www.souzacruz.com.br | Corporate: multinational company | 51 | Brazil | Portuguese |
| Tutun | http://tutun-ctc.md/ | Corporate: non-multinational company | 2 | Ukraine | Romanian |
| Rokok Herbal | www.rokokherbal.com | Corporate: non-multinational company | 1 | Indonesia | Indonesian |
| Guizhou Tobacco Industry Company | www.guiyan.com | Corporate: China National Tobacco Corporation Company | 2 | China | Mandarin |
| Hongyunhonghe Group | www.hyhhgroup.com | Corporate: China National Tobacco Corporation Company | 29 | China | Mandarin |
| Korean Tobacco & Ginseng Corporation | www.ktng.com | Corporate: non-multinational company | 1 | Philippines | English and Korean |
| Style | www.stylesuperslims.ru | Social media brand page: tumblr-style | 1 | Russian Federation | Russian |
| Kiss | www.odnoklassniki.ru/kissclub | Social media brand page: facebook-style | 1 | Russian Federation | Russian, English, Belarusian, Ukrainian , Armenian, Romanian (MD), Romanian (RO), Georgian, Kazakh, Uzbek, Azerbaijani, Turkish |
| Kiss | www.vkontakte.ru/club23459755 | Social media brand page: facebook-style | 6 | Russian Federation, Vietnam | 89 language options |
| 21 Bek | www.odnoklassniki.ru/ru21wek | Social media brand page: facebook-style | 1 | Russian Federation | Russian, English, Belarusian, Ukrainian , Armenian, Romanian (MD), Romanian (RO), Georgian, Kazakh, Uzbek, Azerbaijani, Turkish |
| Djarum Black | www.blackXperience.com | Social media brand page: tumblr-style | 10 | Indonesia | Indonesian and English |
| Clas Mild | www.clas-mild.com | Brand website: all content visible before registration | 3 | Indonesia | Indonesian |
| Senator | www.richmond-tobacco.com | Brand website: limited content visible on registration page | 24 | Russian Federation, Ukraine, Vietnam, Indonesia | English |
| Winston | www.winston.ge | Brand website: limited content visible on registration page | 1 | Turkey | Georgian, English |
| Bond Street | www.bondstreet.ru | Brand website: limited content visible on registration page | 10 | Russian Federation | Russian |
| Kent | www.kent.ru | Brand website: limited content visible on registration page | 4 | Russian Federation | Russian |
| L&M | www.lmlab.ru | Brand website: limited content visible on registration page | 9 | Russian Federation | Russian |
| Marlboro | www.marlboro.ua | Brand website: limited content visible on registration page | 1 | Ukraine | Ukrainian, English |
| British American Tobacco: Lucky Strike | www.pefc.org | Non-tobacco website: Programme for the Enforcement of Forest Certification website | 1 | Pakistan | English |
| Philip Morris (Thailand) Ltd: Marlboro | www.mhlw.go.jp/topics/tobacco/main.html | Non-tobacco website: Japanese Ministry of Health website | 2 | Thailand | Japanese, English |
| **Not included in coding** |  |  |  |  |  |
| More | www.be-more.ru | Brand website: no content visible before registration | 7 | Russian Federation | Russian |
| Russkiy Stil | www.clubrs.ru | Brand website: no content visible before registration | 3 | Russian Federation | Russian |
| Mevius and Mild Seven | www.designinmotion.ru | Brand website: no content visible before registration | 5 | Russian Federation | Russian |
| Camel | www.discovermore.ru | Brand website: no content visible before registration | 18 | Russian Federation | Russian |
| Glamour | www.glamour-life.ru | Brand website: no content visible before registration | 12 | Russian Federation | Russian |
| Kiss | www.kiss-club.ru | Brand website: no content visible before registration | 18 | Russian Federation, Vietnam | Russian |
| LD | www.LD-info.com | Brand website: no content visible before registration | 14 | Russian Federation | Russian |
| Chesterfield | www.mychesterfield.ru | Brand website: no content visible before registration | 4 | Russian Federation | Russian |
| Petr 1 | www.petr-1.ru | Brand website: no content visible before registration | 7 | Russian Federation | Russian |
| Sobranie | www.sobranie.ru | Brand website: no content visible before registration | 12 | Russian Federation | Russian |
| West | www.westonline.ru | Brand website: no content visible before registration | 5 | Russian Federation | Russian |
| Wings | www.wings.ru | Brand website: no content visible before registration | 2 | Russian Federation | Russian |
| Winston | www.winston.ru | Brand website: no content visible before registration | 16 | Russian Federation | Russian |
| Maxim | www.maxim-info.org | Brand website: no content visible before registration | 3 | Russian Federation | Russian |
| Habana Cigarettes | www.cigarrilloscubanos.com.mx | Corporate website: only partially active | 4 | Mexico | Spanish |
| PT KTP Karya Tajinan Prima Malang | www.cakragkn.com | Corporate website: only partially active | 1 | Indonesia | Indonesian and Latin (filler text) |
